# Co-expression of CMTM6 and PD-L1: a novel prognostic indicator of gastric cancer

**DOI:** 10.1186/s12935-020-01734-6

**Published:** 2021-01-28

**Authors:** Chao Zhang, Shutao Zhao, Xudong Wang

**Affiliations:** Department of Gastrointestinal Nutrition and Hernia Surgery, The Second Hospital of Jilin University, Changchun, 130000 China

**Keywords:** Gastric cancer, CMTM6, PD-L1, Survival

## Abstract

**Background:**

CKLF Like MARVEL Transmembrane Domain Containing 6 (CMTM6) is involved in the epigenetic regulation of genes and tumorigenesis. Programmed cell death ligand 1 (PD-L1) is closely related to the prognosis of some human cancers. CMTM6 is a key regulator of PD-L1 in many cancers. The purpose of this study was to investigate the expressions of these proteins in gastric cancer and the correlations with clinicopathological features and survival.

**Methods:**

The expression levels of CMTM6 and PD-L1 were examined in 185 gastric cancer specimens using immunohistochemistry, quantitative real-time PCR and Western blot. Immunofluorescence was used to examine the localizations of CMTM6 and PD-L1. Chi-square test was used to analyze the relationship between CMTM6 and PD-L1 expressions and clinicopathological characteristics. Kaplan–Meier method and log-rank test were used to analyze the survival data of patients.

**Results:**

The positive expression rates of CMTM6 and PD-L1 in gastric cancers were 78.38% (145/185) and 75.68% (140/185), respectively. CMTM6 and PD-L1 were both mainly expressed in the cell membrane and nucleus of gastric cancer tumor cells. High expression of CMTM6 and PD-L1 was correlated with Borrmann type (*P* < 0.001), N stage (*P* = 0.002), peritoneal metastasis (*P* = 0.007) and TNM stage (*P* = 0.038). CMTM6 and PD-L1 expression in gastric cancer tissues showed a positive correlation (Pearson’s coefficient test, r = 0.260; *P* < 0.001). CMTM6 may positively regulate PD-L1 expression. High expression of CMTM6 was correlated with poor prognosis of gastric cancer patients (HR = 1.668; 95% CI = 1.032–2.695; *P* = 0.037). High expression of both CMTM6 and PD-L1 may be an independent factor for overall survival (HR = 1.554; 95% CI = 1.011–2.389; *P* = 0.044).

**Conclusion:**

The combined detection of CMTM6 and PD-L1 may be used as an indicator for judging the prognosis of gastric cancer patients.

## Background

In 2020, 27,600 new cases of gastric cancer (GC) were diagnosed in the United States, which resulted in 11,010 deaths [1]. In 2014, the incidence of GC in China was 30 in 100,000 cases, while the mortality rate was 21.48 in 100,000 [2]. Because of the lack of markers for early detection of GC, it is mostly diagnosed in the late stage, and these patients are not eligible for radical resection. Furthermore, GC tumors are not sensitive to chemotherapy, which leads to higher mortality and poor prognosis of these patients [3]. Therefore, identification of key markers and effective therapeutic targets for the prevention and treatment of GC is critical.

CKLF Like MARVEL Transmembrane Domain Containing 6 (CMTM6) is a member of a protein family that is encoded by different gene clusters on chromosome 16 (CMTM1–4) and chromosome 3 (CMTM6–8). CMTM6 contains a MARVEL (MAL and related proteins for vesicle trafficking and membrane link) region as well as four transmembrane structures. CMTM6 plays a key role in the trafficking of transmembrane proteins and secretory proteins. CMTM6 can also activate and chemotax a large number of immune cells and affects the proliferation and invasion of tumor cells [4–7].

Programmed death ligand-1 (PD-L1) is a negative immunoregulator that inhibits the activation of T cells and induces the apoptosis of anti-tumor T cells. The immunosuppressive tumor microenvironment enables tumor cells to evade the body’s immune response and disables the body’s anti-tumor mechanisms, which increases the occurrence and development of various tumors [8–12]. However, 70% of cancer patients do not respond well to anti-PD-L1 immunotherapy, which indicates the need to identify new therapeutic targets or to develop a combination of immune agents for improved treatment [13].

CMTM6 and PD-L1 are co-localized on the plasma membrane and circulating endosomes. As a regulatory molecule of PD-L1, CMTM6 enhances the cell surface expression of PD-L1. Previous studies showed that CMTM6 and PD-L1 are involved in tumor promoting pathways [14]. However, few studies have examined the functions of CMTM6 and PD-L1 in GC.

In this study, we examined the expressions of CMTM6 and PD-L1 in GC tissues by immunohistochemistry and the correlations with clinicopathological features with the aim of exploring new combination immunotherapy treatments for GC.

## Methods

### Patients

A total of 185 GC tissue specimens from radical gastrectomy were obtained in our center from March 2009 to June 2012. Another 30 pairs of adjacent tissues were included as controls. The age of the 185 GC patients ranged from 39 to 78 years, with a median age of 60 years; the patient group included 126 men and 59 women. Of all patients, 61 had a tumor diameter < 5 cm and 124 had tumors with a diameter ≥ 5 cm; 15 showed high differentiation, 34 medium differentiation, and 136 poor differentiation. Tumor site distribution was as follows: upper, 24 cases; middle, 26 cases; and lower, 135 cases. A total of 143 cases were Borrmann I, II or III, while 42 cases were Borrmann IV. Restaging using the AJCC eighth edition TNM staging revealed 18 cases in stage I, 24 cases in stage II, 132 cases in stage III, and 11 cases in stage IV. Twenty-eight patients had peritoneal metastasis and 157 had no metastasis. All the cases were single lesions, and the patients did not receive chemoradiotherapy before surgery. The postoperative follow-up data were complete, and the median follow-up time was 2.8 years. The study protocol was approved by the hospital ethics committee, and all patients signed informed consent.

### Immunohistochemical staining

The specimens preserved in the pathology department were fixed with 10% neutral formalin and embedded in paraffin. The tissue specimens were sliced in a thickness of 4 μm. After dewaxing and hydration, slides were incubated in EDTA antigen repair solution (pH 9) at 121℃ for 10 min. The slides were immersed in 3% H_2_O_2_ solution and soaked at room temperature for 10 min to eliminate endoperoxidase. Samples were incubated with primary antibodies against CMTM6 (1:100, Abcam, Cambridge, MA, USA) and PD-L1 (1:200; Cell Signaling Technology, Danvers, MA, USA) at 4℃ overnight. Samples were then incubated with secondary antibodies at 37℃ for 30 min and then stained with 3, 3′-diaminobenzidine, counterstained with hematoxylin for 1 min, 1% hydrochloric acid alcohol differentiation, tap water flushed back to blue for 10 min, dehydration in gradient ethanol, and transparent xylene intervention. Samples were observed with a mounting microscope.

### Scoring of immunoreactivities

We scored CMTM6 and PD-L1 staining based on staining intensity and the proportion of positive cells. The staining intensity scores were as follows: 0 for colorless, 1 for yellow, 2 for brown, and 3 for dark brown. The proportions of positive cells were scored as follows: 0 for 0%–5%, 1 for 5%–25%, 2 for 25%–50%, 3 for 50%–75%, and 4 for > 75%. The final score was the product of the staining intensity and the percentage of positive cell scores. We classified cases with a score ≥ 5 as positive expression, and cases with a score of ≤ 4 as negative expression.

### Immunofluorescence assay

Tissue sections underwent double immunofluorescence staining. The staining for the first antibody was performed according to the immunohistochemical procedure. On the following day, samples incubated with fluorescent secondary antibody. DAPI was used to stain the nucleus, and images were obtained using a fluorescence microscope.

### Cell culture

The immortalized cell line GES-1 and gastric cancer cell lines SGC-7901, MGC-803, HGC-27 were cultured in DMEM or RPMI1640 medium containing 10% serum. Cells were cultured in an incubator containing 5% CO_2_.

### Small interfering RNA (siRNA) transfection

Cells plated in 6-well plates were transfected with negative control or the siRNA targeting CMTM6 (GenePharma, Shanghai, China) when the cells were 80% confluent. Cells were collected for analysis 48 h later. The siRNA sequences were as follows: si-CMTM6 sequence: si-CMTM6-1, 5′-CCTCACTGAGCCACTTAAT-3′ and si-CMTM6-2, 5′ CCCTCACTGAGCCACTTAA-3′.

### Quantitative real-time PCR (qRT-PCR)

RNA was extracted from cells using Trizol (Invitrogen, Carlsbad, CA, USA) according to the manufacturer’s instructions. cDNA was synthesized by a reverse transcription kit (Takara, Tokyo, Japan). The PCR instrument was used for reaction amplification. GAPDH mRNA was used as an internal control, gene expression was determined, as relative expression level = 2^−ΔΔCt^ value.

### Western blot

Cells were lysed using RIPA lysis buffer. Equal amounts of protein (30 μg) were separated by electrophoresis and transferred to a PVDF membrane. The membrane was incubated with 5% skim milk at room temperature for 2 h. Membranes were incubated with primary antibodies against CMTM6 (1:1000, Cell Signaling Technology, Danvers, MA, USA) and PD-L1 (1:1000, Cell Signaling Technology) overnight at 4 °C. On the following day, the membrane was incubated with secondary antibody. High sensitivity enhanced chemiluminescence (ECL) was used to detect the target band.

### Cell fractionation assays

Follow the instructions of the nuclear and cytoplasmic protein extraction kit (Invitrogen, Carlsbad, CA, USA). Resuspend the cells in Buffer A and centrifuged at 2000 rpm at 4 °C for 30 s. The supernatant is the cytoplasmic protein. Aspirate the remaining supernatant in the original tube, resuspend the cell pellet in 1 ml buffer A, centrifuged at 12000 rpm at 4 ℃ for 30 s, discard the supernatant, added buffer B, and centrifuged at 12000 rpm at 4℃ for 20 min. The supernatant is the nuclear protein. Finally, Western blot experiment was carried out.

### Co-immunoprecipitation

SGC-7901 cell total lysates were incubated with primary antibody (IgG as control) and Protein A agarose beads at 4ºC for 8 h. After washes with PBS buffer, samples were analyzed by Western blot.

### Statistical analyses

The relationship between the expression of CMTM6 and PD-L1 and the clinicopathological factors was analyzed using SPSS 21.0 software. The correlation between the expression of CMTM6 and PD-L1 was analyzed using the Pearson test. The survival analysis was performed using the Kaplan–Meier method and the survival difference among the different groups was calculated by the log-rank test. Comparisons of the two groups were performed using* t* test. A single factor and a multi-factor Cox proportional hazard ratio model were fitted. *P* < 0.05 was considered to indicate statistical significance.

## Results

### The relationship between the expression of CMTM6 and PD-L1 and clinicopathological factors in GC

**Fig. 1:**
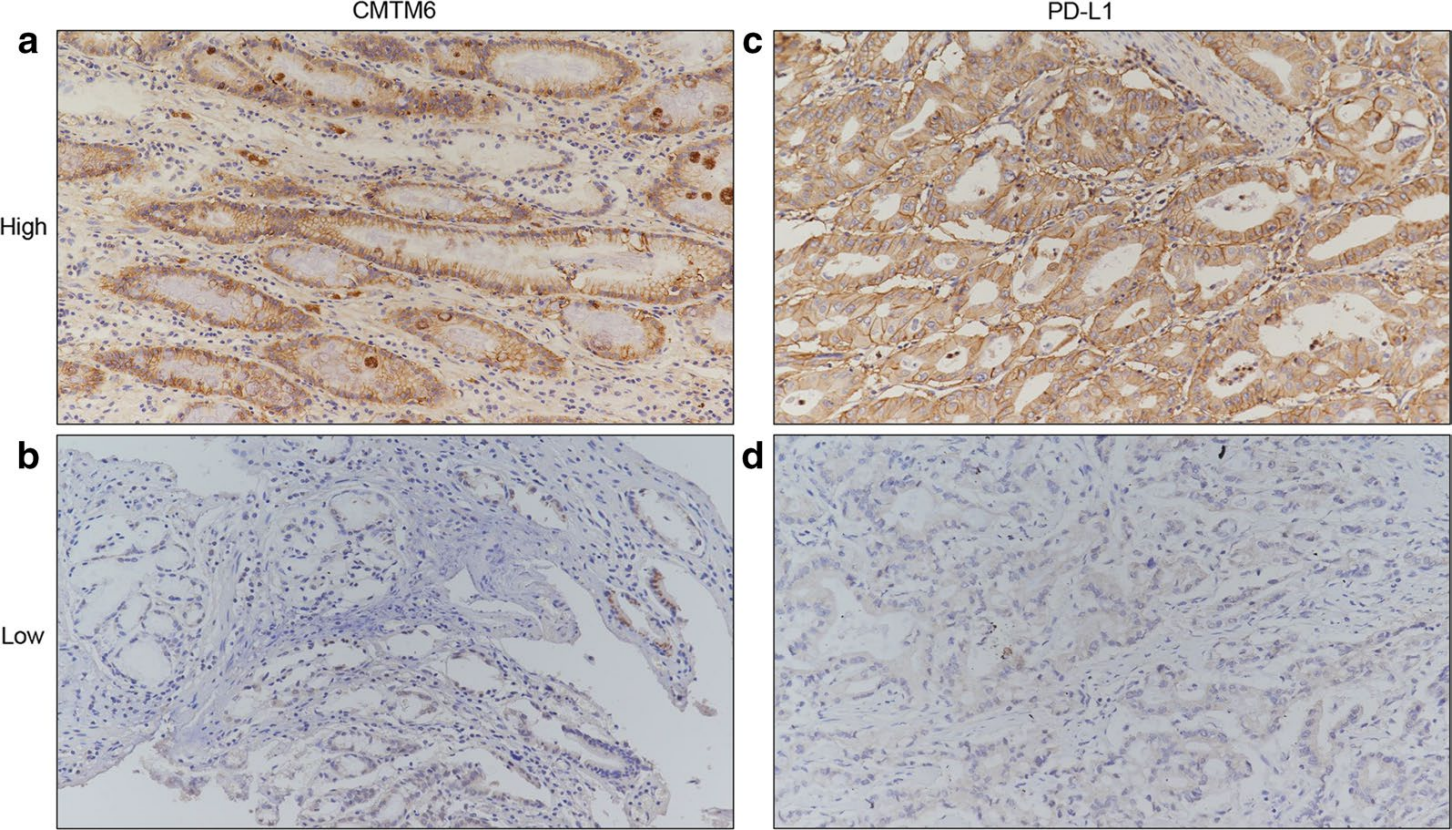
Representative images of immunohistochemical staining for CMTM6 and PD-L1 from patients with gastric cancer. **a** High expression of CMTM6. **b** Low expression of CMTM6. **c** High expression of PD-L1. **d** Low expression of PD-L1. Original magnification at ×20

**Fig. 2:**
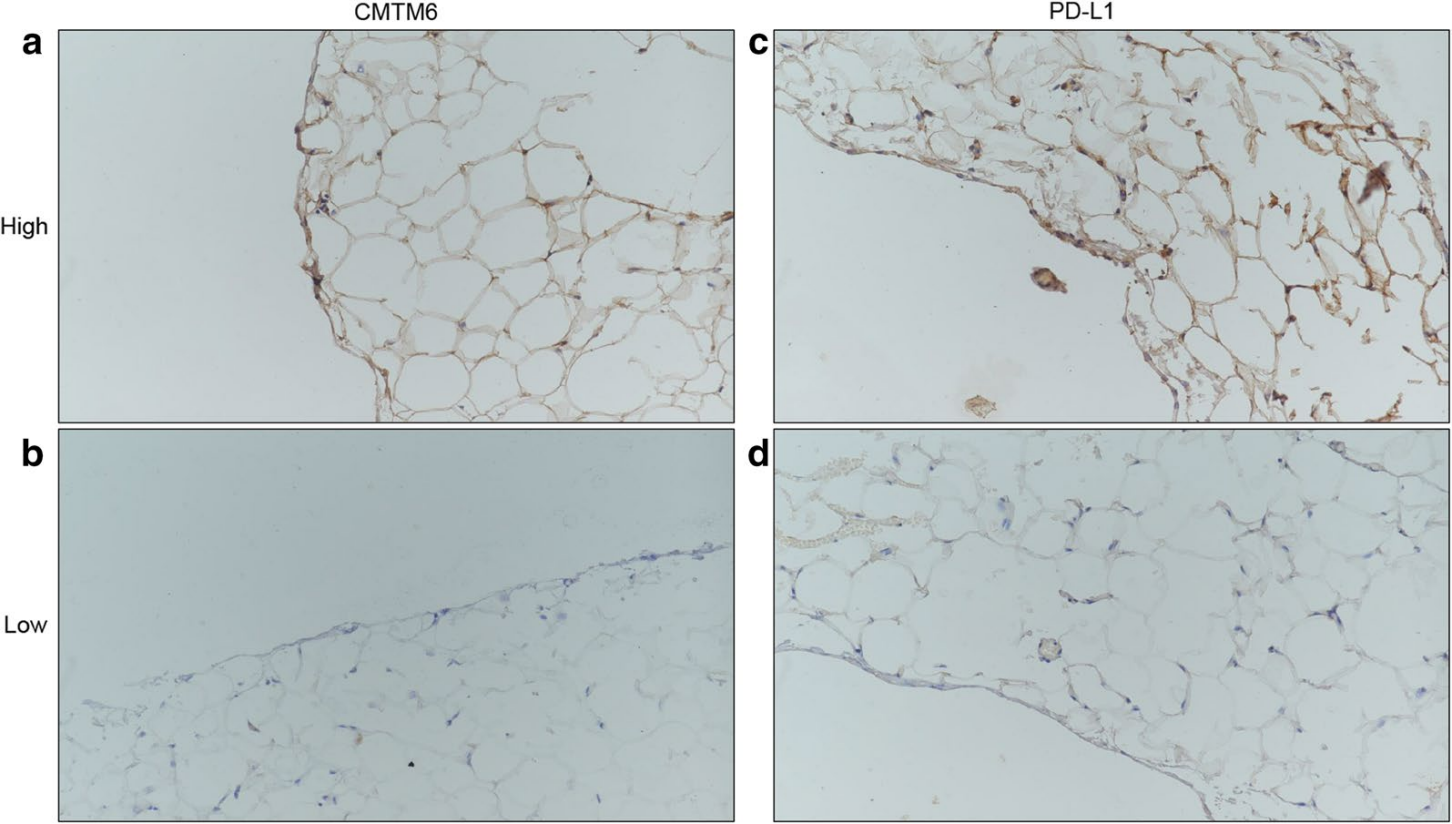
Representative images of immunohistochemical staining for CMTM6 and PD-L1 from patients with gastric cancer peritoneum tissues. **a** High expression of CMTM6. **b** Low expression of CMTM6. **c** High expression of PD-L1. **d** Low expression of PD-L1. Original magnification at ×20

**Fig. 3:**
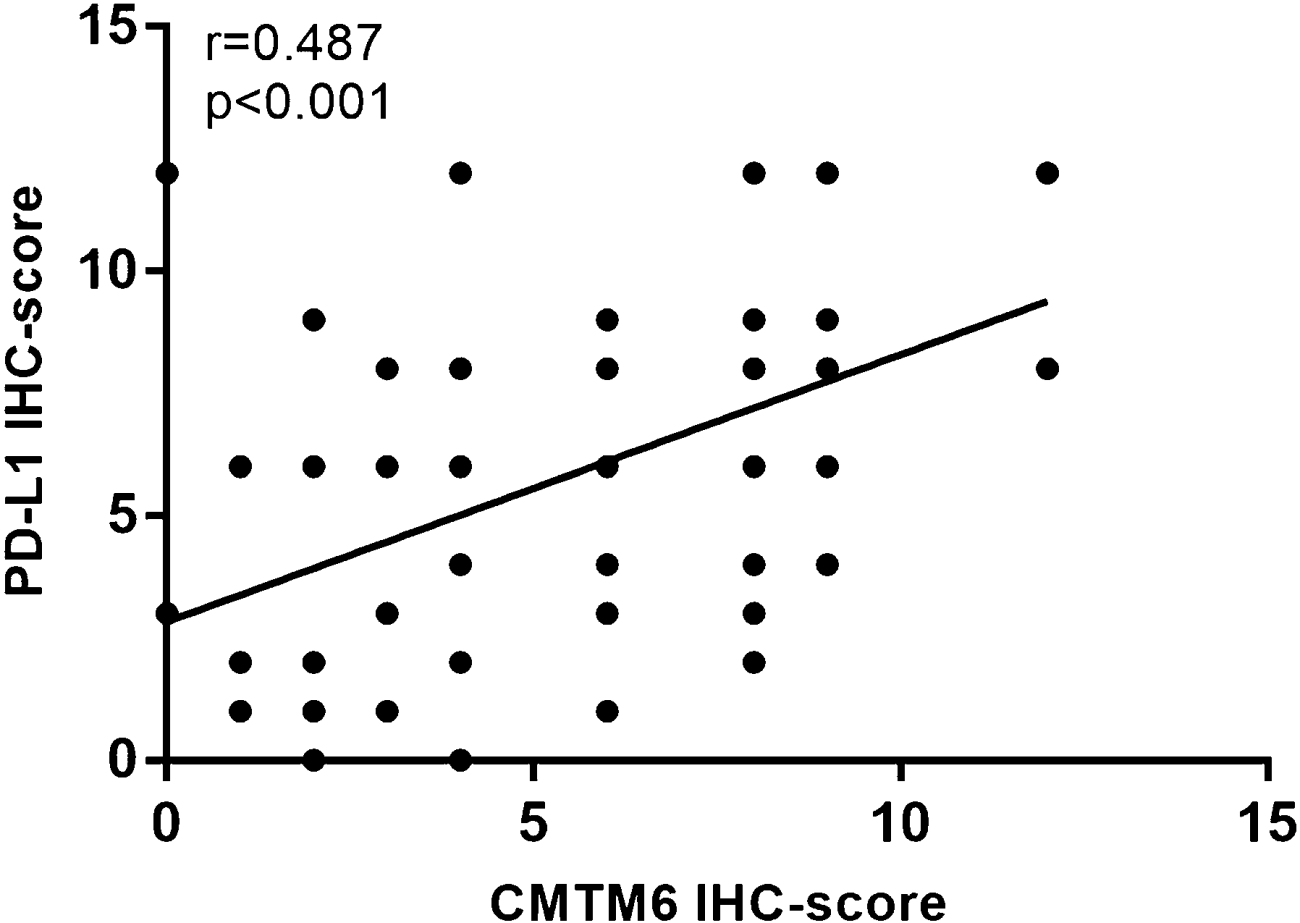
Scatter plot for correlation analysis of the expression level between CMTM6 and PD-L1 in gastric cancer

Immunohistochemical staining results showed that CMTM6 and PD-L1 were mainly expressed in the cytoplasm and cell membrane in GC tissues and peritoneal tissues (Figs. 1, 2). The positive rates of CMTM6 and PD-L1 expression in GC tissues were 78.37% (145/185) and 75.68% (140/185), respectively. There was a positive correlation between CMTM6 expression and PD-L1 expression in GC (Pearson test, r = 0.487; *P* < 0.001, Fig. 3).

The relationship between CMTM6 and PD-L1 and clinicopathological factors is shown in Table 1. High expression of CMTM6 and PD-L1 was significantly correlated with Borrmann type, lymph node metastasis, peritoneal metastasis, and TNM staging (all *P* values were < 0.05). Conversely, the expressions of CMTM6 and PD-L1 were not related to patient age, sex, tumor size, tumor differentiation, tumor location, or T stage.

**Table 1:** Relationship between the CMTM6 and PD-L1 expression and clinical parameter

Variables	CMTM6 expression		*P *value	PD-L1 expression		*P *value
	High (n = 145)	Low (n = 40)		High (n = 140)	Low (n = 45)	
Age			0.750			0.637
< 60	72	21		69	24	
≥ 60	73	19		71	21	
Sex						
Male	102	24	0.214	93	33	0.387
Female	43	16		47	12	
Size						
< 5 (cm)	51	10	0.226	45	16	0.672
≥ 5 (cm)	94	30		95	29	
Differentiation						
Well	12	3	0.748	10	5	0.468
Moderate	25	9		28	6	
Poor	108	28		102	34	
Tumor location						
Upper	18	6	0.670	15	9	0.251
Middle	19	7		21	5	
Lower	108	27		104	31	
Borrmann type						
III	110	33	< 0.001	111	32	< 0.001
IV	35	7		30	12	
Invasive depth						
T1	1	1	0.359	2	0	0.645
T2	24	9		25	8	
T3	17	7		16	8	
T4	103	23		96	30	
lymph node metastasis						
N0	27	16	0.005	25	18	0.002
N1-N3	118	24		115	27	
Peritoneal recurrence (M)						
M0	119	38	0.043	113	42	0.007
M1	26	2		27	1	
Stage (TNM)						
I	10	8	0.035	9	9	0.038
II	20	4		19	5	
III	105	27		102	29	
IV	10	1		10	1	
PD-L1 expression						
Low	27	18	< 0.001			
High	118	22				

*CMTM6* CKLF Like MARVEL Transmembrane Domain Containing 6, *PD-L1* Programmed death ligand-1

### Relationship between the expression of CMTM6 and PD-L1 and prognosis

**Fig. 4:**
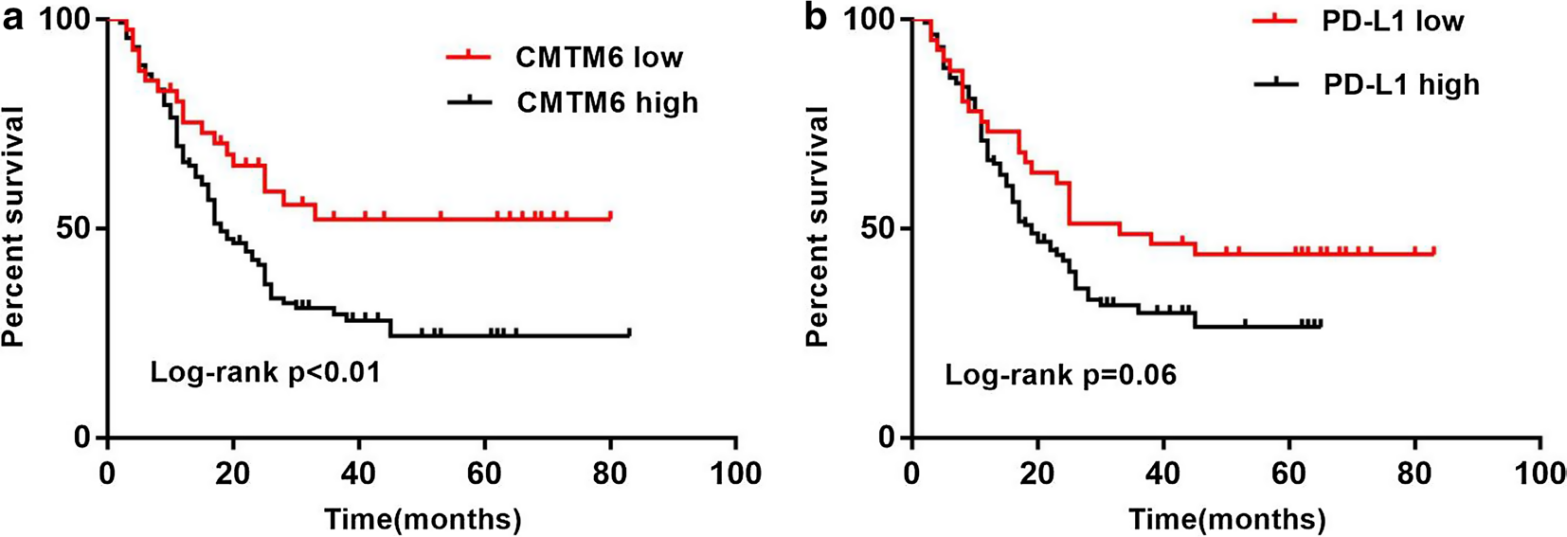
Kaplan–Meier survival curves. **a** CMTM6. **b** PD-L1

Of the 185 patients with GC, 77 (41.62%) were still alive at the time of writing; the median overall survival (OS) was 16 months (range 0–83 months). The 5-year survival rate of positive CMTM6 was 52.27% vs. 24.31% (*P* < 0.01) and that of positive PD-L1 was 43.77% vs. 26.46% (*P* > 0.05). Survival analysis showed that high CMTM6 expression was associated with shorter OS, while high PD-L1 expression showed no significant effect on OS (Fig. 4).

In univariate analysis, Borrmann type (IV; HR = 2.164, 95% CI 1.444–3.241, *P* < 0.001), T stage (HR = 2.627, 95% CI 1.327–4.889, *P* = 0.003), lymph node metastasis (HR = 2.829, 95% CI 1.475–5.424, *P* = 0.002), peritoneal recurrence (HR = 1.919, 95% CI 1.2633–2.914, *P* = 0.002), high expression of CMTM6 (HR = 1.668, 95% CI 1.032–2.695, *P* = 0.037), and high expression of CMTM6 combined with high expression of PD-L1 (HR = 1.757, 95% CI 1.162–2.655, *P* = 0.007) were associated with OS. Cox multivariate analysis further confirmed that Borrmann (IV) type, N stage, peritoneal metastasis, and CMTM6 combined with PD-L1 were independent prognostic factors of OS (HR = 1.891, 95% CI 1.248–2.864, *P* = 0.003; HR = 2.313, 95% CI 1.187–4.505, *P* = 0.014; HR = 1.941, 95% CI 1.274–2.957, *P* = 0.002; HR = 1.554, 95% CI 1.011–2.389, *P* = 0.044, respectively; Table 2). High expression of CMTM6 was associated with poor prognosis, while patients with high expression of both CMTM6 and PD-L1 showed worse OS (HR = 2.120; 95% CI = 1.618–4.020; *P* = 0.021; Table 3). Our results further demonstrated that the combined expression of both PD-L1 and CMTM6 may be more suitable as a prognostic indicator than CMTM6 alone.

**Table 2:** The univariate and multivariate analyses of factors associated with overall survival

Variable	univariate Cox regression			multivariate Cox regression		
	HR	95%CI	P-value	HR	95%CI	P-value
Age (≥ 60)	1.224	0.839–1.785	0.295			
Sex (male)	1.016	0.677–1.526	0.937			
Size (≥ 5 cm)	1.678	1.085–2.595	0.02			
Differentiation (Poor)	0.773	0.504–1.185	0.773			
Tumor location (Middle low)	1.176	0.670–2.065	0.572			
Borrmann type (IV)	2.164	1.444–3.241	0.000	1.825	1.202–2.770	0.005
Invasive depth T3-T4	2.627	1.327–4.889	0.003			
lymph node metastasis ( +)	2.829	1.475–5.424	0.002	2.395	1.228–4.671	0.010
Peritoneal recurrence ( +)	1.919	1.263–2.914	0.002	1.704	1.102–2.637	0.017
High CMTM6 expression	1.668	1.032–2.695	0.037			
High PD-L1 expression	1.457	0.922–2.301	0.107			
CMTM6 + / PD-L1 +	1.757	1.162–2.655	0.007	1.554	1.011–2.389	0.044

*CMTM6* CKLF Like MARVEL Transmembrane Domain Containing 6, *PD-L1* Programmed death ligand-1

**Table 3:** Survival analyses by subgroups for gastric cancer patients according to the Cox proportional hazards model

Variable	High CMTM6			High PD-L1		
	HR	95%CI	P-value	HR	95%CI	P-value
CMTM6						
Low				0.937	0.396–2.219	0.883
High				1.816	1.006–3.277	0.048
PD-L1						
Low	1.231	0.546–2.774	0.617			
High	2.120	1.618–4.020	0.021			

*CMTM6* CKLF Like MARVEL Transmembrane Domain Containing 6, *PD-L1* Programmed death ligand-1

### CMTM6 and PD-L1 colocalization

**Fig. 5:**
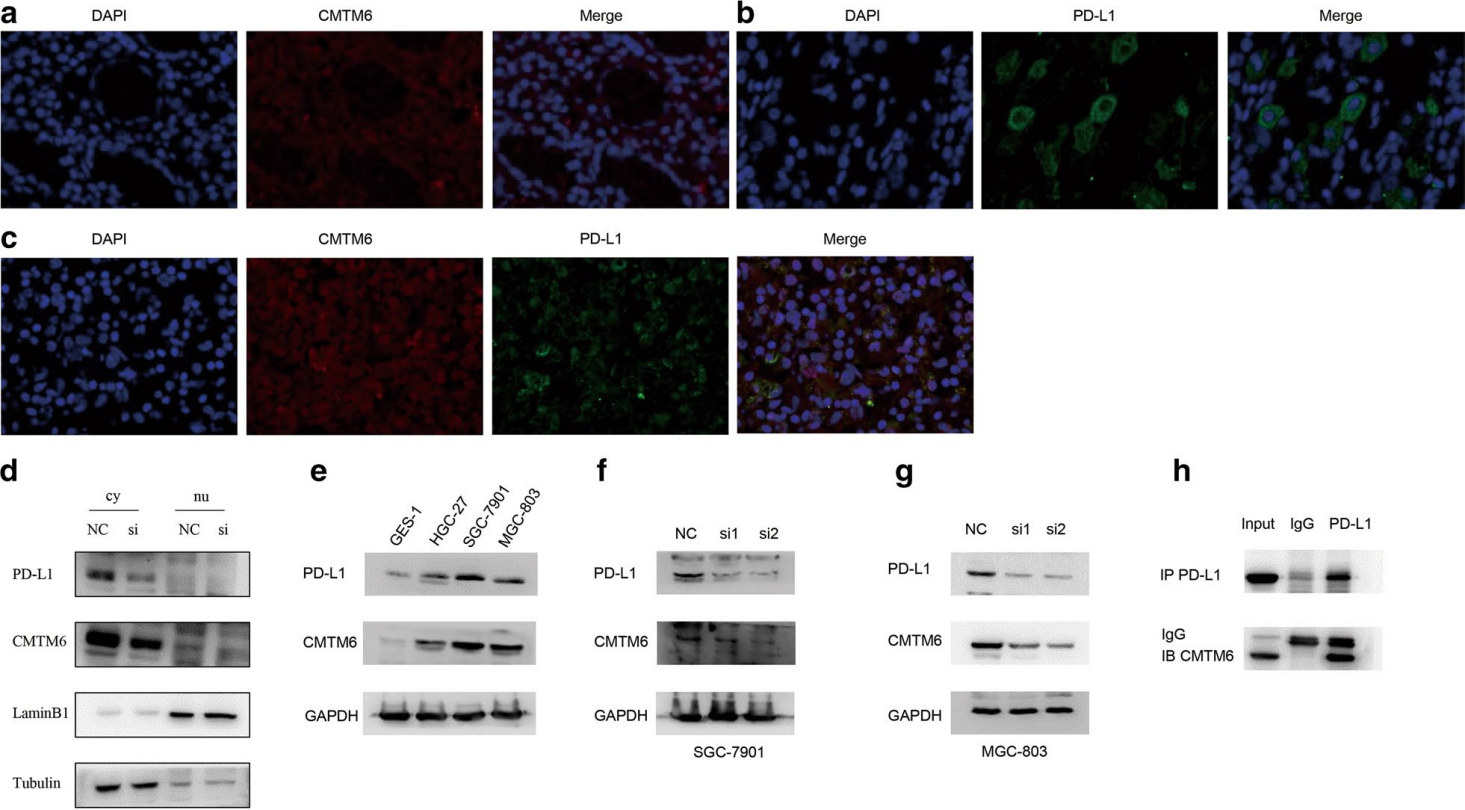
Immunofluorescence, Cell fractionation assays, Co-immunoprecipitation, Western blot, and small interfering RNA (siRNA) transfection analyses for gastric cancer cell lines. **a** Immunofluorescence analysis of gastric cancer tissues for CMTM6. Red indicates CMTM6, blue indicates the nuclei. **b** Immunofluorescence analysis of gastric cancer tissues for PD-L1. Green indicates PD-L1, blue indicates the nuclei. **c** Immunofluorescence analysis of gastric cancer tissues for CMTM6 and PD-L1. Red indicates CMTM6, green indicates PD-L1, blue indicates the nuclei. **d** Nuclear and cytoplasm distribution of CMTM6 and PD-L1. **e** Western blot analysis for gastric cancer cell lines. **f**, **g** Knockdown of CMTM6 in SGC-7901 and MGC-803 cells. **h** co-IP of CMTM6 and PD-L1

CMTM6 was localized in the cell membrane and cytoplasm of GC tissues, labeled as bright red fluorescence; Notably, PD-L1 was also expressed in the cell membrane and cytoplasm of GC tissues, labeled as bright green fluorescence (Fig. 5a, b, c). In order to further verify the cell localization of CMTM6 and PD-L1 in SGC-7901 cells, we confirmed that CMTM6 and PD-L1 were mainly expressed in the cell membrane and cytoplasm through the cell fractionation assays (Fig. 5d).

### CMTM6 regulates the expression of PD-L1

We examined the expression levels of CMTM6 and PD-L1 in cell lines and found that the expression levels of CMTM6 and PD-L1 in cancer cell lines were higher than levels in GES-1 cells (Fig. 5e, Supplementary file, Figure S1,2). High CMTM6 expression was detected in SGC-7901, MGC-803 cells, and these cell lines were selected to generate cells with small interfering-mediated knock-down of CMTM6. We found that the expression of PD-L1 was decreased after CMTM6 was knocked down, which indicates that CMTM6 may regulate the expression of PD-L1 (Fig. 5f, g, Additional file 1: Figure S3,4). We performed co-IP experiments and found that CMTM6 binds to PD-L1 (Fig. 5h).

## Discussion

Here we analyzed the expression of CMTM6 and PD-L1 in 185 GC tissues by immunohistochemistry and examined the associations with clinicopathological characteristics and survival of GC patients. We found that expression of CMTM6 or PD-L1 alone was not an independent prognostic factor in patients with GC after excluding other confounding factors. Co-expression of CMTM6 and PD-L1 was an independent prognostic factor in GC patients.

In our study, 15.1% of patients had peritoneal metastasis. The reason for surgical treatment is that the imaging and physical signs of the patients are not manifested, and micrometastasis was found during the operation. N stage and M stage correlated with OS, and T stage was not an independent prognostic factor; this may be because of the high percentage of lymph node metastasis regardless of T stage. Borrmann type IV GC shows a specific biological behavior with a high degree of malignancy and accounts for 10%–20% of all GC; the 5-year survival rate of this cancer type is only 0%–17%. In our study, Borrmann type IV accounted for 22.7% of cases, closing to the highest proportion. Bowman type IV GC indicates lymph node metastasis, more common peritoneal metastasis, and late staging during surgery [15]. Borrmann type IV was also an independent prognostic factor in our study.

CMTM6 plays different roles in different cancers. Guan et al. analyzed CGGA, TCGA and other databases and found that CMTM6 was highly expressed in glioblastoma multiforme and mesenchymal subtypes, and high expression of CMTM6 was related to poor prognosis [5]. Cox model analysis showed that CMTM6 was an independent prognostic factor of glioma, which indicated that CMTM6 played an important role in tumor invasion and progression. Zhu et al. found that the expression of CMTM6 in hepatocellular carcinoma was significantly lower than that in adjacent non-tumor tissues through immunohistochemical detection, and the prognosis of cases with low CMTM6 expression was better [16]. One possible mechanism is that CMTM6 binds with PD-L1 protein, decreases its ubiquitination and increases the half-life of PD-L1 protein, resulting in enhanced ability of tumor cells to inhibit T cells; the elimination of CMTM6 would reduce PD-L1 and improve OS. The conclusion of this study was different from that of Zhu et al. Our survival analysis showed that the OS of patients with high expression of CMTM6 was poor, which may be due to the difference of CMTM6 expression in different cancers. The specific reasons need to be further explored. However, our results suggest that CMTM6 may be a new immune checkpoint molecule.

As an immunosuppressive molecule, PD-L1 can inhibit the activity of T cells through a variety of complex signaling pathways, thus promoting tumor progression [17–19]. The relationship between the expression of PD-L1 and the prognosis of different cancer patients has been controversial. A meta-analysis study involving 7308 digestive system cancer patients found that high expression of PD-L1 was associated with poor prognosis (HR = 1.44, 95% CI 1.18–1.76, *P* < 0.001), especially in GC (HR = 1.43, 95% CI 1.05–1.94, *P* = 0.021) [20]. Böger et al. suggested that high expression of PD-L1 was associated with good prognosis (HR = 0.753, 95% CI 0.584–0.971, *P* = 0.029) [21]. Our study also found that PD-L1 overexpression was not associated with poor prognosis, and this may be related to the difference in sample size and sample selection. Despite the rapid development of immune checkpoint blockade, a large proportion of patients still fail to benefit from anti- PD-L1 immunotherapy. Therefore, Das et al. [22] proposed a strategy for finding new immune checkpoints and adopting a combination of multiple immune checkpoint blockers.

CMTM6 has been established as another important immune checkpoint and regulates the anti-tumor immune effect mediated by T lymphocytes. However, whether CMTM6 can regulate PD-L1 and what role it plays in GC has not been studied. Our immunohistochemical results show that the expression of CMTM6 is positively correlated with the expression of PD-L1, and the expression level of CMTM6 and PD-L1 increases with the increase of malignant degree of GC. Through immunofluorescence and cell fractionation assays, we found that CMTM6 and PD-L1 were mainly co-localized in the cell membrane and cytoplasm of GC tumor cells, and the expression of CMTM6 and PD-L1 in GC cell lines was higher compared with levels in GES-1 cells. We silenced the expression of CMTM6 in SGC-7901 and MGC-803 cells and found that the expression of PD-L1 also decreased, which suggested that CMTM6 may positively regulate the expression of PD-L1 in GC cells. This indicates that the regulation of CMTM6 and PD-L1 signaling pathway in the tumor microenvironment has a synergistic effect. Based on previous studies and our results, we speculate that CMTM6 may activate the transmission of related signals in the PD-L1 pathway or enhance the secretion of some cytokines in the tumor immune response, thus promoting the progression of GC. Mezzadra et al. [23] found that CMTM6 can enhance the ability of tumor cells expressing PD-L1 to inhibit T cells. The elimination of CMTM6 can decrease the expression of PD-L1 and then significantly reduce the inhibition of tumor-specific T cell activity, but the specific regulatory mechanism needs to be further studied. Another important finding is that the prognosis of patients with high expression of CMTM6 was poor, and in cases in which PD-L1 was also highly expressed along with high expression of CMTM6, these patients show a worse prognosis. Burr et al. [13] found that CMTM6 through binding to PD-L1 directly regulates anti-tumour immunity. We also verified this conclusion in gastric cancer through co-IP experiment. Our findings suggest that PD-L1 depends on CMTM6 to perform its inhibitory function, and that the combination of high expression of CMTM6 and PD-L1 may be more suitable as a marker of GC than the individual proteins. Whether CMTM6 can be combined with PD-L1 monoclonal antibody inhibitors as a new target for immunotherapy of GC will become a research focus in the future [24, 25].

There are still some limitations to our study. Our study is a retrospective study, which may have certain selection bias. However, it is the first to study the clinicopathological correlation between CMTM6 and PD-L1 in GC; these findings may provide the experimental basis for the formation of dual-targeting drugs.

## Conclusion

We found that CMTM6 with high expression is related to poor prognosis, and the prognosis is worse when PD-L1 is also highly expressed. The combination of CMTM6 and PD-L1 immune agents may open up a new strategy for immunotherapy.

We are the first to study the significance of combined detection and application of CMTM6 and PD-L1, which provides a basis for future research of dual-targeting drugs and is of great significance for future immunotherapy.

## Supplementary information

**Additional file 1: Figure S1.** qRT-PCR analysis for gastric cancer cell. A CMTM6 expression level in cell lines. B PD-L1 expression level in cell lines. C, D Knockdown of CMTM6 in SGC-7901 and MGC-803 cells.

## Data Availability

Please contact author for data requests.
