# Supplementary figures and images for: Co-expression of CMTM6 and PD-L1: a novel prognostic indicator of gastric cancer
Source: Cancer Cell Int. 2021 Jan 28;21:78. doi: 10.1186/s12935-020-01734-6 (PMC7842018; doi:10.1186/s12935-020-01734-6)

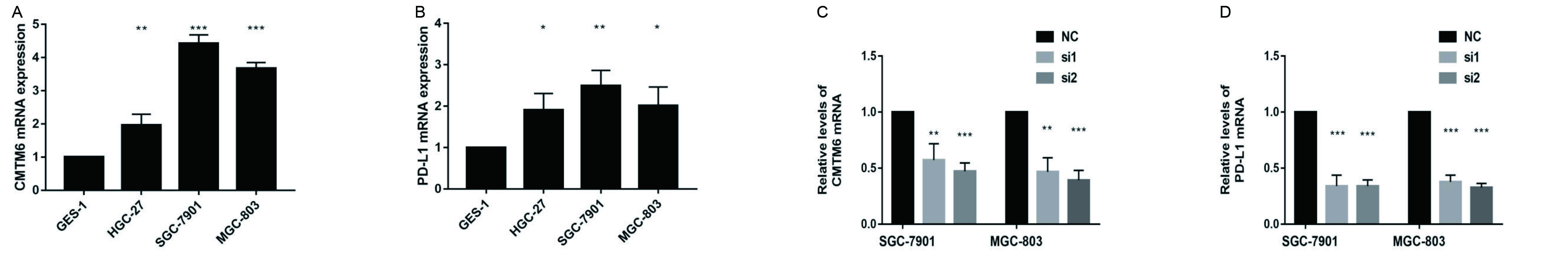

Supplement: Supplementary file 1 — Additional file 1: Figure S1. qRT-PCR analysis for gastric cancer cell. A CMTM6 expression level in cell lines. B PD-L1 expression level in cell lines. C, D Knockdown of CMTM6 in SGC-7901 and MGC-803 cells. [file 12935_2020_1734_MOESM1_ESM.jpg]
